# QUALITY OF LIFE OF OLDER ADULTS DURING THE COVID-19 PANDEMIC: RACIAL DISPARITIES AND SOCIAL CONNECTEDNESS

**DOI:** 10.1093/geroni/igad104.2729

**Published:** 2023-12-21

**Authors:** Babatope Ogunjesa, Sadia Anjum Ashrafi, Rafael Guimaraes, Andiara Schwingel

**Affiliations:** University of Illinois--Urbana-Champaign, Champaign, Illinois, United States; University of Illinois Urbana Champaign, Urbana, Illinois, United States; University of Illinois at Urbana-Champaign, Champaign, Illinois, United States; University of Illinois, Urbana Champaign, Champaign, Illinois, United States

## Abstract

Social connectedness is crucial for well-being, especially among older adults. The Covid-19 pandemic has worsened mental distress in society due to prolonged social isolation practices aimed at curbing the virus’s spread. This study examined disparities in older adults’ self-reported quality of life during the pandemic and the influence of family and friends interaction. This study utilizes secondary data from the coronavirus worries and social isolation among older adults study by AP-NORC Center for Public Affairs Research on individuals aged 50 years and above (N=1,051). We aim to observe the effect of social interactions on the self-reported quality of life (QoL) of adults. Logistic regression analysis and marginal effect post-estimation analysis were utilized in this study, with adjustments made for race, education, gender, and income. The result showed that individuals who meet with family and friends more often (AOR=17.19, 95% CI=3.75-78.88, p< 0.05), or about as often (AOR=2.24, 95% CI=1.15-4.36, p< 0.05), are more likely to report a high quality of life than those who meet with them less frequently. A marginal effect analysis revealed that White adults had an 89.5% probability of reporting a high quality of life, non-Hispanic Black adults had an 83.4% probability of high quality of life, Hispanic adults had an 88.3% probability of high quality of life, and individuals from other ethnic groups had an 83.3% probability of high quality of life. These findings underscore the need for targeted interventions that address social isolation, especially among older adults, particularly those from racial and ethnic minority groups.

